# Bacteriophage-Derived Vectors for Targeted Cancer Gene Therapy

**DOI:** 10.3390/v7010268

**Published:** 2015-01-19

**Authors:** Md Zahidul Islam Pranjol, Amin Hajitou

**Affiliations:** 1 Institute of Clinical and Biomedical Science, University of Exeter Medical School, Exeter, Devon EX1 2LU, UK; E-Mail: z.pranjol@exeter.ac.uk; 2 Phage Therapy Group, Department of Medicine, Burlington Danes Building, Imperial College London, Hammersmith Hospital, Du Cane Road, London W12 0NN, UK

**Keywords:** bacteriophage, CMV, Grp78, AAVP, *HSVtk*, RGD4C-AAVP/*CMV-HSVtk*, RGD4C-AAVP/*Grp78-HSVtk*, glioblastoma

## Abstract

Cancer gene therapy expanded and reached its pinnacle in research in the last decade. Both viral and non-viral vectors have entered clinical trials, and significant successes have been achieved. However, a systemic administration of a vector, illustrating safe, efficient, and targeted gene delivery to solid tumors has proven to be a major challenge. In this review, we summarize the current progress and challenges in the targeted gene therapy of cancer. Moreover, we highlight the recent developments of bacteriophage-derived vectors and their contributions in targeting cancer with therapeutic genes following systemic administration.

## 1. Introduction

### 1.1. Overview of Gene Therapy and Its Historical Perspective

Gene therapy describes the delivery of a functional therapeutic gene to target cells, which may be used to knockdown expression of a particular macromolecule, over-express a desired protein, directly induce cell death, or replace a defective or mutant gene to allow expression of a normal protein product. The concept of gene therapy arose in the early 1960s, as Joshua Lederberg conceived of the idea of a direct control of nucleotide sequences in human chromosomes, coupled with recognition, selection, and integration of the desired gene [1]. He was the first to mention the grafting of polynucleotide sequences onto a virus by chemical procedures. In the mid- to late-1960s, scientists showed the transformation of normal cells to a neoplastic phenotype by covalently, stably, and heritably integrating genetic information of the papovavirus SV40 into the genomes of target cells [2]. Subsequently came the recombinant DNA era, which provided a promising platform for developing efficient methods of gene transfer and specific genes in cloned forms. However, it was not until 1990, when the first clinical study using gene transfer was reported, whereby a retroviral vector (RV) was used to transfer the neomycin resistance marker gene into tumor-infiltrating lymphocytes obtained from five patients with metastatic melanoma [3].

Although originally conceived to treat congenital diseases, today, over 66% of clinical trials in gene therapy are designed to treat cancer [4]. Since the developmental scheme of gene therapy began in the 1970s, the practice has expanded into numerous cancer research areas with the ambition of developing a universal anti-cancer vector that can be safely administered, tolerated within normal human physiology, and selectively target tumorigenic cells. Cancer, a highly heterogeneous disease, remains a leading cause of death worldwide, accounting for 7.6 million deaths in 2008 (World Health Organization, WHO). Chemotherapy and radiotherapy are the current treatments against the disease, with chemotherapy remaining the most potent offence. However, poor drug uptake by tumor cells due to therapeutic resistance, high interstitial pressure, and the irregular tumor vasculature, are ever-present challenges which allow cancer cells to find a way to evade even the most effective anti-cancer therapies presently in place. Additionally, systemic administrations of conventional chemotherapeutic treatments against cancer present the risk of contributing to the appearance of secondary tumors.

Recently, several cancer gene therapy vectors have undergone preclinical and clinical trials, and several other vectors have the potential to be tested in the near future. In this review, we aim to summarize the targeting strategies used in cancer gene therapy and the development of eukaryotic delivery vectors with their advantages and disadvantages. Next, we will emphasize the chimeric bacteriophage (phage) vector, named adeno-associated virus/phage (AAVP), as a promising candidate in targeted systemic gene therapy of cancer.

## 2. Cancer Gene Therapy and Specificity

Although numerous gene therapy strategies have been developed to treat cancer, a major challenge has been to generate a systemic gene delivery vector that can both selectively and efficiently target tumor tissue. To date, most clinical trials involved intratumoral injection, of either viral or nonviral vector systems, to avoid transgene expression in normal healthy tissue. A local delivery of the transgene is necessary as proof-of-principle, but systemic administration is clinically beneficial as it permits the vehicle delivery to metastases and some of the primary tumors, which necessitate invasive procedures for local access. Indeed, development of efficient systemic vectors would ensure safety, with only the desired tissues are targeted, while sparing the healthy neighboring cells and organs. The major challenges of clinical gene therapy trials are vector targeting and the high cost of vector production. Therefore, the development of targeted vectors, which mediate efficient and long-term expression of the therapeutic gene in tumor tissues after systemic administration, should provide a major advancement in cancer gene therapy. To date, two main approaches have been used for tumor targeting: (i) transcriptional targeting, which uses promoters that are only active in the target tumors, and (ii) ligand-targeting of vectors to specific receptors expressed within the tumor tissue [5]. Each of these approaches has been attempted individually, by numerous studies, and showed promising results for vector delivery and transgene expression in preclinical tumor models.

### 2.1. Transcriptional Targeting

Selective targeting and killing of tumor cells is a major goal of current cancer gene therapy. Placing a transgene under the influence of a cell-type-specific promoter raises the chance of its expression in that particular cell [6]. Usually these promoters are highly expressed in certain tissues (malignant tumor) and remain at a low basal expression level in normal tissues, which can reduce or even eliminate potential toxic side effects of the therapeutic gene in these normal tissues. The use of promoters, such as *cytomegalovirus* (CMV) promoter in adenoviral (Ad) vectors based gene therapy, has been well characterized for its constitutive activity, both *in vitro* and *in vivo*. However, the promoter itself does not significantly discriminate between cell types, rather, is expressed at high levels in a range of mammalian tissues [7], which is problematic in cancer gene therapy. On the other hand, promoters, which are active in tumor cells, have only been used for targeted gene delivery to tumors, in order to selectively deliver transgene expression to the tumor tissue.

Transcriptional targeting was first reported by two studies in 1997. Rodriguez *et al.* restricted transgene expression in prostate-specific antigen (PSA) producing prostate cells by applying the prostate-specific antigen promoter into adenovirus type 5 DNA to drive transgene expression, which they referred to as “attenuated replication-competent adenovirus” [8]. Moreover, using the albumin promoter, replication of the herpes simplex virus type 1 (HSV-1) vector was restricted to albumin-expressing liver cells [9]. Since then, numerous promoters have been characterized and used in targeted cancer gene therapy (Table 1).

#### 2.1.1. Tissue-Specific Promoter

This group of promoters is active and mediates transgene expression in only specific tissues. Several tissue-specific promoters that target tumors of a single origin were characterized and used in cancer gene therapy [10]. Examples include the ovarian-specific promoter to target ovarian cancer [11], the albumin promoter to target hepatocellular carcinoma [12], and the thyroglobulin promoter for thyroid carcinomas [13]. The tyrosine kinase promoter has been used, both *in vitro* and *in vivo*, to target melanomas [14].

**Table 1:** Examples of ligand and transcriptional targeting used in eukaryotic viral vectors in targeted cancer therapy.

Vector	Tumor-Specific Promoters	Ligand Targeting	References
Ad	PSA, GH, TRE, rTG, AFP, VEGFR-2, flt-1, hTERT	RGD, NGR, SIGYPLP, CGKRK, SIKVAV	[13,15,16,17,18,19]
AAV	PRC1, RRM2, BIRC5	NGR, GFE	[20,21]
HSV-1	Albumin, ANGPTL-3, E2F-1	ND *	[9,22]
LV	PSA/E	αCD20, hSCF	[17,23]
RV	CEA, GRP78, kdr, E-selectin	ND *	[17,24,25]
MV	ND *	HSNS, HAA	[26]

AAV: adeno-associated virus; ANGPTL-3: human angiopoietin-like 3; BIRC5: Baculoviral IAP repeat-containing 5; E2F-1: transcription factor 1; GH: Growth hormone; PSA/E: Prostate-specific antigen/Enhancer; hSCF: human stem cell factor; HSNS/HAA: modified attachment protein H on MV; LV: lentivirus; MV: measles virus vector; PRC1: protein regulator of cytokinesis 1; RRM2: ribonucleotide reductase subunit 2; rTG: rat thyroglobulin; SIGYPLP, CGKRK, SIKVAV: homing peptides; RV: retrovirus; TRE: Tetracyclin response element; VEGFR2: Vascular endothelial growth factor receptor 2; * ND: Not determined.

The prostate-specific antigen (PSA) promoter was used in targeting prostate cancer. PSA is predominantly expressed in prostate cells due to transcriptional activation. Both in cell culture and *in vivo*, PSA promoter has been previously shown to express the herpes simplex virus thymidine kinase (*HSVtk*) suicide gene in PSA-positive prostate cancer cells and prostate tumors, respectively; however, no transgene expression was observed in cells that do not express PSA [27].

Although these promoters proved efficient to deliver transgene expression in tumor cells, their activity, in both normal, as well as tumor cells, is deemed to be a major drawback.

#### 2.1.2. Tumor-Specific Promoter

Tumor-specific promoters constitute an ideal choice for targeted cancer gene therapy in order to direct the expression of therapeutic genes, as they have been shown to be highly active in tumor cells while having little or no activity in normal cells. Based on their characteristics, tumor-specific promoters have been subdivided into four groups [10]: (i) cancer-specific promoters; (ii) tumor-type-specific promoters; (iii) tumor vasculature-related promoters; and (iv) tumor microenvironment-related promoters.

##### Cancer-Specific Promoters

Cancer-specific promoters, such as the promoter of the telomerase gene, are active specifically in malignant cells and have the great potential in cancer gene therapy to target a wide variety of tumors. Telomerase is highly active in around 85%–90% of human cancer cells, while remaining either low or undetectable in normal tissues [28]. Telomerase is composed of two active subunits, telomerase RNA (hTR) and catalytic component human telomerase reverse transcriptase (hTERT). The promoters of these two subunits are highly active in telomerase-positive cells, such as tumor and fetal cells. Thus, these two promoters have been individually used in many targeted cancer gene therapy studies to drive therapeutic gene expression, demonstrating enhanced killing of telomerase-positive cells. Although the *hTR* or *hTERT* promoters have been broadly utilized for transcriptional regulation of therapeutic genes, they still have some limitations in clinical use as they possess low activity and some potential toxicity to certain normal cells has also been reported [15].

##### Tumor-Type-Specific Promoter

Tumor-type-specific promoters are the promoters of oncofetal genes that are often overexpressed in certain types of tumors and are silent in normal tissues. The most well-characterized promoters of this group include α-fetoprotein (AFP) promoter that is active in fetal liver and hepatocellular carcinomas [29], and carcinoembryonic antigen (CEA) promoter, which is active in a proportion of breast, lung, colorectal and pancreatic cancers. This promoter was extensively used in different vector systems to selectively deliver various therapeutic genes, such as cytosine deaminase or *HSVtk* expression in CEA-positive cells, and the results demonstrated significant tumor growth suppression or regression, with no toxicity to liver and other normal organs, following prodrug 5-fluorocytosine or ganciclovir (GCV) administration, respectively [30]. Although these promoters mediate transgene expression in tumor tissues, and may, therefore, be good candidates for transcriptional targeting in cancer gene therapy, their application still remains limited since they cannot be administered for a variety of tumors.

##### Tumor Vasculature-Related Promoters

The genes encoding this group of promoters are overexpressed in proliferating endothelia tumor microvasculature. Examples of such promoters that have been used in targeted gene therapy are E-selectin and endothelial-specific kinase insert domain receptor (KDR/flk-1) which are upregulated in tumor endothelium. The promoters of these genes efficiently expressed tumor necrosis factor-α (TNF-α) by 10-fold increase in endothelioma cells compared to non-endothelioma cells [24]. In addition, the promoters of genes encoding Flt-1, vascular endothelial growth factor receptor 1, and human preproendothelin-1 have also been used in gene therapy vector systems and proved efficient to drive transgene expression in the vasculature of tumors and metastases [16]. While the usage of these promoters in targeted cancer gene therapy proved efficient to deliver transgene expression in the tumor vasculature, they still have limitations as some of these promoters were shown to be active in small vessels and upregulated in injured vessels as well [31].

##### Tumor Microenvironment-Related Promoters

Tumor microenvironment-related promoters belong to the genes that are upregulated in response to the tumor microenvironment and physiology. Compared to normal cells, tumor cells demonstrate high growth rate and an increased glucose metabolism. Moreover, neovascularization and angiogenesis may fail to keep pace with tumor growth which creates a “cancer microenvironment”-hypoxia, acidosis and glucose deprivation-characterizing poorly vascularized solid tumors. As a part of the cancer cell’s response to adapt to these conditions, the promoters of some genes such as hypoxia response elements (HREs) and some of the heat shock family genes become induced.

Recently, the heat shock genes, such as the gene of the *Glucose regulated protein 78* (Grp78), have gained increasing interest in targeted cancer gene therapy because of their activation in a wide variety of tumors. The activity of its promoter and its ability to drive transgene expression within areas of tumor hypoxia, which are highly resistant to current forms of treatment, makes it even a more attractive promoter to use in targeted cancer gene therapy. Indeed, therapeutic transgene expression driven by this promoter is induced in response to insufficient blood supply and tumor necrosis and reached high levels leading to complete tumor eradication in preclinical models [32].

For cancer targeting gene therapy, the Grp78 promoter seems to be an ideal promoter to restrict expression of the therapeutic gene within the tumor tissue, and is, therefore, worthwhile a critical evaluation.

## 3. Grp78 as an Endogenous Macromolecule in Cancer

Grp78 gene encodes a 78-kDa protein (Grp78) that acts as an endoplasmic reticulum (ER) stress response chaperone. It has 60% amino acid homology to the 70-kDa heat shock protein (HSP70), and, hence, Grp78 has been categorized within the HSP70 family. Despite this homology, Grp78 is not induced by heat stress and it also primarily localizes in the endoplasmic reticulum (ER) lumen to function as a molecular chaperone in an ATP-dependent manner. Recently, however, this protein was shown to be present in the cytoplasm and expressed on the cell surface membranes; thus, it can be utilized as a biomarker for stressed cells, such as in tumors [33].

Also known as immunoglobulin heavy chain binding protein (BiP), Grp78 orchestrates unfolded protein response (UPR) by binding to unfolded, misfolded, and incorrectly glycosylated proteins in the ER lumen. In eukaryotic cells, when the protein production exceeds the folding capacity of the ER, the misfolded proteins elicit UPR. Under normal conditions, Grp78 remains bound to three transmembrane sensor proteins: protein kinase-like ER kinase (PERK), inositol requiring enzyme 1 (IRE1), and activating transcription factor 6 (ATF6). As the threshold of the incorrectly folded protein load exceeds a certain level, Grp78 dissociates from the sensors and binds these proteins. This dissociation process leads to activation of a signaling cascade, UPR. The outcome is a decrease in biosynthetic burden of the ER by desensitizing the cells to ER stress and ultimately upregulating pro-survival genes via transcriptional activation in the nucleus, such as Grp78 promoter to elevate Grp78 expression [33]. If, however, the ER homeostasis cannot be re-established, the UPR induces programed cell death [34].

Tumor microenvironment and lack of pace of neovascularization and angiogenesis result in an uncontrolled production of mutant and misfolded ER proteins that lead to the accumulation of unfolded or misfolded ER proteins, which subsequently trigger the UPR. Thus, while Grp78 expression remains low in major adult organs, such as brain, heart, and lung, it is highly upregulated in transformed cells and in several tumors, such as glioblastoma (GBM), breast cancer, and prostate cancer [35], and in the endothelia of tumor vasculature [36]. This overexpression of Grp78 has been shown to correlate with an enhanced tumor recurrence risk, tumor grade, and decreased survival rate in cancer patients [34,37].

Grp78 plays a major role in cancer cell survival by activating the pro-survival pathway. *In vivo* studies with heterozygous Grp78 mice (*Grp7^+/−^*) demonstrated marked impeded tumor progression compared to wild-type Grp78 mice, as tumor size was reduced and apoptosis was promoted. In addition to primary tumors, Grp78 levels are highly induced in metastasis and assist secondary tumor survival by maintaining neovascularization [36].

Pyrko and colleagues demonstrated a positive correlation between Grp78 overexpression and glioblastoma (GBM) cell proliferation rate by knockdown of Grp78 in GBM cells that led to a reduced proliferation [38]. In addition to tumor cell survival and proliferation, cytoplasmic Grp78 plays an important role in blocking the apoptosis of stressed cells by binding and inhibiting activation of caspases-7 and -12. Grp78 expressed on several tumor cell surfaces promoted cell proliferation, survival, and metastasis by binding to plasma proteinase inhibitor α2-macroglobulin and activating its downstream signaling pathway [33].

## 4. Grp78 Promoter in Cancer Gene Therapy

The use of Grp78 as a promoter in cancer gene therapy was first proposed by the group of Lee [25]. A retroviral vector construct carrying the *HSVtk* transgene was used to infect tumor cells and subsequently implanted into mice. Administration of GCV, along with *HSVtk* expression, driven by Grp78 promoter, was shown to suppress and eradicate murine and human breast tumor xenografts in mice [32]. Moreover, another group assessed the efficacy of *HSVtk* under the control of Grp78 promoter in gastroesophageal junction and gastric adenocarcinomas cells and reported significant cell death *in vitro* and tumor regression *in vivo* following GCV treatment [39].

In transgenic mice, the *LacZ* transgene, driven by the rat Grp78 promoter, showed high transgene expression in cancer cells, while remaining inactive in major adult organs [32]. In addition, Grp78 gene transcription can increase over time [40] because, unlike viral promoters, such as CMV, mammalian promoters are not silenced in eukaryotic cells, thus, resulting in stronger and long-term transgene expression from the vector.

## 5. Ligand-Directed Targeting Ensures Specific and Efficient Transgene Delivery

Progress in tumor vascular targeting has provided a platform to target agents safely, efficiently and selectively in tumorigenic tissues. The use of *in vivo* phage display screenings has significantly contributed to the identification of such target receptors in the affected tumor endothelium of animal models [41]. As angiogenesis is vital for tumor progression, targeting these tumor-specific and tumor associated endothelial cell-specific receptor molecules holds the potential for ligand-directed targeting in cancer gene therapy (Table 2) [42]. Viral vectors can be engineered to display homing peptides on their surface in order to target specific receptor-bearing cell types within a host. This method of targeting ensures that the vector only infects the cells bearing the receptor, while leaving non-receptor-bearing cells untouched.

It was hypothesized, long ago, that selectively interfering with the tumor blood supply would lead to strong antitumor effects [42]. In addition, vascular cells are readily accessible through the systemic circulation of the vector; hence, targeting and killing tumor vasculature has been of great interest [42]. Neovasculatures that support tumor growth express different markers on their endothelium as compared to normal quiescent ones, such as α_v_β_3_-integrin (adhesion molecule), and an estimated 100 tumor cells are supplied by one endothelial cell [43]. Additionally, several tumorigenic tissues express these integrins, such as glioblastoma (GBM), melanoma, *etc*. This cumulative understanding of the tumor physiology and biochemistry has led to the development of ligand-directed vectors targeting the tumor vasculature via α_v_-integrin receptors by using α_v_-integrin binding ligands that ensure specificity and optimize effectiveness.

**Table 2:** List of receptors used in targeted cancer therapy [42].

Receptor	Function/Class	Localization
Grp78	Stress Response	Tumor cells
αv Integrins	Cell adhesion	EC, tumor cells
CD13	Aminopeptidase N	EC, pericytes
APA	Aminopeptidase A	Pericytes, stromal cell
NG2/HMWMAA	Proteoglycan	Pericytes, tumor cells
MMP-2/MMP-9	Metalloproteinases	EC, tumor cell
HSP90	Heat shock	EC, tumor cells

EC, endothelial cells.

### 5.1. Ligand Targeting of Viral Gene Therapy Vectors

In the last decade several different techniques have been developed with the aim of facilitating vector targeting to specific cells after systemic administration. However, for systemic targeted cancer gene therapy, the choice of an efficient vector system, able to deliver an appropriate expression level of the therapeutic gene in cancer cells, is the most crucial step. In this process, non-viral vectors, which involve physical methods such as a gene gun, magnetofection and chemical methods, such as lipoplexes, inorganic nanoparticles, as well as injection of naked DNA, hold some potential due to their mass production, safety, and low host immunogenicity. However, their low level of transfection and transgene expression still present great challenges [44]. In contrast to non-viral vectors, arguably, viral vectors have proven to be the vectors of choice as they naturally evolved to infect mammalian cells and can mediate high level of transduction (Table 1). Although, as of 2007, several types of vectors were used in 70% of gene therapy clinical trials, many groups are aiming at improving viral vector features for the purpose of gene therapy [45]. Most progress in viral gene therapy has involved the lentivirus (LV), adenovirus (Ad), and adeno-associated virus (AAV) [46]. In particular, Ads have been utilized as an attractive approach for cancer gene therapy and have been reported as the most frequently used vectors in clinical trials [47]. Ads contain 36 kb double-stranded DNA, which provides space for inserting large sequence fragments. In addition to the ease of vector manipulation and the capability to produce high titers, Ad vectors mediate efficient transgene expression in both dividing and non-dividing cells. However, despite all the advantages of Ad vectors, they can induce severe toxicity due to raising host immune response after systemic delivery of the required dose of vector. Thus, for selective killing of tumor cells, many studies have focused on modifying the adenoviral genome in such a way to achieve cancer specificity at different stages through the adenoviral life cycle [48]. One main approach has involved the specific deletion in Ad genome and generation of replication-incompetent Ad vectors which attenuate the viral amplification and, thus, inhibit the infection. These engineered vectors can be further modified to selectively replicate in tumor cells to mediate their lysis. ONYX-015 vector is such an example of a replication-incompetent Ad vector. This vector cannot defend itself against the p53 tumor suppressor gene products and, thus, is destroyed in healthy cells. In contrast, as the p53 gene is defective in most tumor cells, ONYX-015 can proliferate within these tumor cells and mediate their lysis [49]. Moreover, if the tumor lysis mediated by these vectors is not sufficient, therapeutic genes can be inserted into the Ad vector genome in order to enhance the antitumor efficacy [50].

AAVs, member of the parvovirus family, are also promising vectors that have been used in numerous gene therapy applications as they confer low immunogenicity within the host. Moreover, AAVs have shown to mediate stable and long-term gene expression *in vivo* [51]. Additionally, AAV vectors are purified to high titers and, like Ads, can transduce both dividing and non-dividing cells. However, one of the severe limitations of AAV vectors for gene therapy is their limited gene cloning capacity (just over 4 kb) [47]. AAVs contain 4.7 kb single-stranded DNA encoding for two genes: *rep* which is responsible for viral DNA replication and *cap*, which is in charge of packaging the viral genome. *Rep* and *cap* open reading frames (ORFs) are flanked by two inverted terminal repeats (ITRs) [52]. On entry into the host cell, the AAV single stranded DNA is converted to transcriptionally active double-stranded DNA and remains episomal [51]. To generate recombinant AAV vectors, *rep* and *cap* are replaced by transgene cassettes, leaving the ITR cis-elements which serve as origin of replication [52].

Although eukaryotic viral vectors, such as AAV, boast efficient transgene delivery and extremely stable long-term expression of the transgene, their innate, broad tropism for mammalian cells pose the risk of inducing an immune response in the host, which escalates risks of re-administration of the vector. In addition, AAV vectors have limited cloning capacity, as they cannot accommodate large-size cDNA. Moreover, due to the wide tropism for mammalian cells, eukaryotic viral vectors are frequently taken up by the liver, reticulo-endothelial system, and other unwanted tissues after intravenous administration [5].

## 6. Bacteriophage-Guided Gene Therapy of Cancer

**Figure 1:**
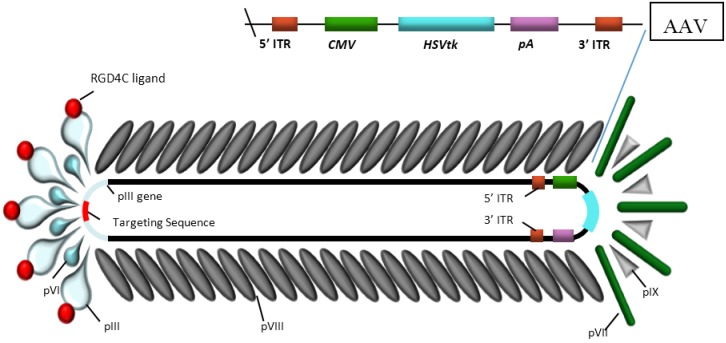
Structure of the hybrid vector AAV/phage (AAVP) developed by Hajitou *et al.* [54]. The particle contains a chimeric genome of a CMV-transgene cassette flanked by inverted terminal repeats, 3' ITR and 5' ITR, of AAV-2 and the genome of M13 filamentous bacteriophage. The outer capsid belongs to the M13 phage and hence lacks tropism for mammalian cells. The capsid contains a major coat protein pVIII and four minor coat proteins pIII, pVI on one side and pVII pIX on the other. The α_v_-integrin binding ligand, RGD4C, is expressed on the pIII minor coat protein of AAVP in order to allow ligand-directed targeting of the tumor vasculature and tumor cells.

Bacteriophages, the viruses that only infect bacteria, present an alternative and safer strategy for targeted systemic delivery of transgenes, as they have no intrinsic tropism for mammalian cells, but can mediate modest gene expression after genetic manipulation. Production of bacteriophages is cost-effective and can be completed at high titers. Moreover, bacteriophages are safe and can be engineered to deliver genes to mammalian cells. Additionally, bacteriophages have been used for antibiotic therapy during the pre-antibiotic era and were safely administered even in children after systemic administration. In 2006, the US Food and Drug Administration (FDA) approved the use of some bacteriophage preparations for ready-to-eat (RTE) meat and poultry products as antibacterial food additives [53]. Most importantly, unlike eukaryotic viruses, bacteriophages do not require further context modification of their capsid as targeting peptides are, in actual fact, selected and isolated directly for targeting specific cell surface receptors after screening of a phage display peptide library. However, they have been, in the past, considered to be poor gene delivery vehicles as they have evolved to infect only bacteria and have no optimized strategies to efficiently express transgenes upon entry into eukaryotic cells [5]. In order to overcome these limitations, Hajitou and colleagues [54] reported a new generation of hybrid prokaryotic-eukaryotic viral vector as a chimera between eukaryotic AAV and the filamentous M13 bacteriophage, then named AAVP (AAV/phage), both contain single stranded DNA genome (Figure 1). This vector expresses three to five copies of the cyclic RGD4C (CDCRGDCFC) ligand on the phage pIII minor coat protein allowing systemic and specific targeting to the α_v_β_3_-integrin receptor, which are expressed primarily on tumor vasculature and tumor cells, and are absent or expressed at barely detectable levels in normal endothelium and tissues [55].

### 6.1. Novel Hybrid Gene Therapy Vector: AAVP

The hybrid vector genome was developed by inserting an engineered AAV (recombinant AAV/rAAV) transgene cassette into an intergenomic region of the phage genome, under the regulation of the CMV promoter and flanked by full-length inverted terminal repeats (ITR) from AAV serotype 2 (Figure 1). The use of AAV ITRs in the targeted AAVP (RGD4C-AAVP) improves transduction efficiency and enhances transgene expression by maintaining and forming concatemers of the eukaryotic transgene cassette [54]. *HSVtk* transgene expression under the CMV promoter (RGD4C-AAVP/*CMV-HSVtk*) and subsequent GCV treatment produces drastic suppression of established tumors in mice and rats.

Since its development, the vector has been under investigation in pre-clinical models. The National Cancer Institute of the USA (NCI) has used the ligand-targeting properties of the RGD4C-AAVP to deliver tumor necrosis factor alpha (TNF-α) to the angiogenic vasculature of human melanoma xenografts in nude mice [56]. In this systemic administration of the phage particle, the TNF-α expression was shown to be specifically localized in tumors, leading to apoptosis in tumor blood vessels and significant inhibition of tumor growth, while remaining virtually undetectable in all other tissues, notably the liver and spleen. However, the RGD4C-AAVP particles, which were found in the latter two vital organs, did not have their transgenes expressed in them. The efficacy of targeted RGD4C-AAVP expressing the TNF-α was assessed in domesticated dogs with soft tissue sarcoma [57]. Intravenous single and multidoses of the vascular-targeted RGD4C-AAVP vector was shown to be tumor specific. Repeated vector administrations resulted in complete eradication of cancer in a few dogs and stability in others, despite the presence of a high immune response against the phage viral particles. Trepel *et al.* showed the presence of a bystander effect after GCV treatment between transduced endothelial cells and non-transduced tumor cells themselves, implying that RGD4C-AAVP/*CMV*-*HSVtk* plus GCV therapy will not be completely limited by transduction efficiency [58]. Although the phage-based particles are known to be immunogenic, repeated administrations in domesticated dogs and immune-competent mice resulted in efficient antitumor therapy [54,57]. The selectivity and safety properties of the RGD4C-AAVP have made this novel, hybrid vector a promising tool that holds great potentials in systemic cancer gene therapy.

### 6.2. Development of the AAVP Viral Particles

#### 6.2.1. Limitations Faced by the CMV Promoter in the Phage: Silencing of Gene Expression

Although the use of the CMV promoter in adenoviral vector-based gene therapy has been well characterized for its constitutive activity both *in vitro* and *in vivo*, the promoter rather remains active at high levels in a range of mammalian tissues. In addition, the silencing of gene expression from the CMV promoter in mammalian cells occurs through several mechanisms, including DNA methylation and histone deacetylation [59]. The CMV promoter activity was reported to be suppressed completely by methylating cytosine resides at 5'CpG dinucleotides within a DNA sequence using Spiroplasma methyltransferase Sssl. Hypermethylation is a phenomenon that is observed in many cancers and affects genes that regulate cell cycle (p16^INK4a^, p15^INK4a^), DNA repair (BRCA1, MGMT), apoptosis (DAPK, TMS1), drug resistance, angiogenesis and metastasis [60]. The propensity of cancer cells towards extensive methylation may, in part, play a role in CMV methylation.

Histone deacetylation is another mechanism of CMV silencing as it ultimately leads to condensed, transcriptionally-inactive regions of chromatin. Chromatin repression and transcriptional remodeling of the *LacZ* gene in HeLa cells transduced with rAAV-*CMV-*LacZ construct occurred upon removal of the histone deacetylase inhibitor, trichostatin-A [61]. These, and other, data suggest that CMV promoter silencing over time can be an obstacle to viral vector-based gene therapy by reducing desired transgene expression in mammalian cells. This necessitates the development of a vector that demonstrates long-term transgene expression and increased systemic anti-tumor efficacy.

#### 6.2.2. A Double-Targeted Phage Vector with the Grp78 Promoter

Combining the two tumor targeting strategies by using tumor homing ligands and tumor specific promoters, in one vector system is challenging, but would provide a major advance in targeted gene therapy of cancer. In 2012, our group has developed a double-targeted AAVP phage particle, whereby both ligand-directed, with RGD4C, and transcriptional targeting strategies were integrated into a single phage vector platform [40]. The transcriptional targeting feature of this novel phage has been achieved by substituting the CMV viral promoter by the Grp78 tumor specific promoter in the AAV transgene cassette. Grp78 is an endogenous macromolecule, which is over-expressed in several tumor cells and, therefore, the ligand-targeted AAVP carrying this promoter should only be expressed in the targeted tumor vasculature and tumor cells.

Kia and colleagues showed long-term transgene expression from AAVP under the regulation of the rat Grp78 promoter in glioblastoma cell lines, compared to CMV promoter which underwent gene silencing over time. Flow cytometry analysis of the *green fluorescent protein* (GFP) expression under Grp78 regulation, in RGD4C-AAVP/*Grp78-GFP* transduced 9L cells*,* showed a drastic increase from 57% (39 days post-vector transduction) to 85% (97 days post-vector transduction). In contrast, only 37% of cells transduced by the RGD4C-AAVP/*CMV-GFP* expressed GFP at day 39, followed by a substantial drop to 11% on day 97. Significantly higher tumor cell killing over time was also observed by *HSVtk*/GCV therapy under Grp78 promoter compared to CMV, both *in vitro* and *in vivo*. *In vivo* studies revealed that when tumors grew back after therapy, repeated GCV treatment resulted in tumor growth inhibition in mice that received the RGD4C-AAVP/*Grp78-HSVtk*. However, little or no effect on tumors was observed in RGD4C-AAVP/*CMV-HSVtk* administered mice. Therapeutically RGD4C-AAVP/*Grp78-HSVtk* was shown to be advantageous over RGD4C-AAVP/*CMV-HSVtk* by producing a marked regression of the large tumors. *HSVtk*/GCV therapy was also shown to activate both promoters of endogenous Grp78 and of the RGD4C-AAVP/*Grp78-HSVtk* vector in Western blot analyses.

#### 6.2.3. Current Development: Inhibition of Histone Deacetylation and DNA Methylation Restore Gene Expression under the CMV Promoter and Enhance Grp78-Regulated Gene Expression

Inhibition of histone deacetylation restored gene expression from the CMV promoter in human (U87) and rat (9L) GBM cell lines transduced with RGD4C-AAVP/*CMV* vector [62]. It was previously reported that histone deacetylases (HDACs) are upregulated in cancer. HDAC class I and II inhibitors trichostatin-A and suberoylanilide hydroxamic acid combined with RGD4C-AAVP carrying CMV or Grp78 promoter reactivated RGD4C-AAVP/*CMV* efficacy and enhanced RGD4C-AAVP/*Grp78* in cancer cells specifically, respectively.

Extensive methylation of the CMV promoter sequences has previously been reported [63], which consequently reduces gene expression. The DNA methylation inhibitor 5-Azacytidine reinstated this phenomenon in rat 9L GBM cell line carrying RGD4C-AAVP/*CMV*, although no significant difference was observed in cells transduced with RGD4C-AAVP/*Grp78*. These are important findings in progressive gene expression under the two promoters in the long-term, and which should be carefully considered for any future clinical applications.

## 7. Conclusions

Cancer gene therapy is a promising approach in treating cancer. However, a vector that has the potential to overcome the decade-long challenges posed by the lack of specificity and the risk of cytotoxicity for healthy cells after systemic administration is crucial. RGD4C-AAVP, with its transcriptional- and ligand-directed-targeting features and stable long-term gene expression under the eukaryotic Grp78 promoter, is a promising tool in targeted systemic cancer gene therapy, and should be brought forth in future clinical trials in cancer patients.
